# Editorial: Reducing feed-food competition: a transdisciplinary approach

**DOI:** 10.3389/fvets.2026.1826205

**Published:** 2026-04-08

**Authors:** Domenico Pietro Lo Fiego, Claudio Forte, Massimo Trabalza-Marinucci, Davide Pravettoni, Antonio Natalello

**Affiliations:** 1 Department of Life Sciences, University of Modena and Reggio Emilia, Reggio Emilia, Italy; 2 Department of Veterinary Sciences, University of Turin, Turin, Italy; 3 Department of Veterinary Medicine, University of Perugia, Perugia, Italy; 4 Department of Veterinary Medicine and Animal Sciences, University of Milan, Lodi, Italy; 5 Department of Agriculture, Food and Environment (Di3A), University of Catania, Catania, Italy

**Keywords:** agro-industrial by-products, circular economy, climate change, former foodstuffs, livestock sustainability

The intensification of livestock production and the rising global demand for animal-source foods have brought increasing attention to issues of environmental sustainability, resource-use efficiency, and food security. Within this context, feed-food competition namely the competing use of agricultural land and resources to produce food directly for humans or to produce feed for livestock (1) has become a central topic in the debate on the future of sustainable agri-food systems. Traditional livestock production, especially monogastric systems, often relies on human-edible crops such as cereals and legumes, potentially increasing direct competition with human nutrition (2). However, more recent analyses show that a substantial proportion of livestock feed could consists of non-edible by-products, which reduces direct competition and promotes more circular and resource-efficient production models (3). Furthermore, livestock can convert inedible biomass into high-quality nutrients, contributing to food security through the supply of proteins and essential micronutrients (4). Assessing feed-food competition thus requires an integrated framework that considers environmental impacts, resource-use efficiency, nutritional contributions, and socio-economic dimensions (5, 6).

The aim of this Research Topic was to collect global evidence on strategies that mitigate feed-food competition through the adoption of sustainable, circular, and transdisciplinary approaches. The nine articles included address two complementary pathways: the incorporation of agro-industrial by-products or former foodstuffs (FFs') into animal diets, and the use of technologies and nutritional strategies aimed at improving feed efficiency. Together, these studies provide valuable insights for researchers, feed manufacturers, farmers, and policymakers.

A first group of studies focuses on the valorization of by-products rich in bioactive compounds. Ferlisi et al. examined the dietary inclusion of an olive mill wastewater phenolic extract (0, 74, or 225 ppm) in 135 heavy pigs. While growth performance, pubertal status, backfat thickness, and histopathological parameters remained unaffected, the highest supplementation level increased hepatic and muscle antioxidant activity and enhanced several serum antioxidant markers. Meat quality analyses revealed reduced cooking loss and changes in color parameters in supplemented pigs, suggesting that dietary polyphenols may influence meat structure and water-holding capacity.

Liu et al. evaluated fermented (FCP) and unfermented citrus pomace (UFCP) in broiler diets. The FCP increased final body weight, average daily gain, and pH_45min_, ${\text{b}}_{24\text{h}}^{*}$, intramuscular fat of breast muscle, and showed a tendency to reduce feed-to-gain ratio. Both CP diets enhanced antioxidant status in serum and breast muscle, increased polyunsaturated fatty acid content, and upregulated genes related to lipid metabolism and antioxidant defenses. Given the overall improvements, the authors support the inclusion of FCP in broiler nutrition, although optimal dosage requires further study.

Li et al. investigated Rosa roxburghii Tratt residue, a local available fruit by-product in Guizhou Province, China, into the diets of 96 Hu sheep at 0, 10, 20, and 30% inclusion. Growth performance and feed efficiency were unchanged, while the 30% diet achieved the highest gross profit. Plasma metabolites showed moderate changes, such as reduced albumin and creatinine and increased HDL levels. Meat quality was only slightly modified, with reduced water-holding capacity and increased shear force at 20 and 30% inclusion levels. Overall, up to 30% residue can be used without compromising performance, offering economic advantages and modest metabolic benefits, with minimal impact on meat quality.

A second thematic area concerns technologies and nutritional strategies that improve feed efficiency (FE), a key factor in sustainable production. Since gut microbiota plays a major role in nutrient utilization, Wang et al. analyzed caecal microbiota and metabolites in 290 Duroc gilts with divergent residual feed intake (RFI). Using 10 low-RFI and 10 high-RFI gilts, the authors found that in low-RFI microbial taxa such as *Escherichia, Eubacterium*, and *Bacteroides* may influence serotonin metabolism, suggesting that the RFI may be partly achieved through tryptophan metabolism in gut microbes. In animals with low RFI, gut microbes may enhance feed efficiency by enhancing host synthesis and metabolism of tryptophan-related metabolites.

Long et al. compared degradation kinetics and intestinal protein digestibility of tomato pomace (TP), whole tomato (WT), and soybean meal (SBM) in cannulated Boer goats. WT showed the highest degradation rates and effective degradability for dry matter, organic matter, crude protein, and fiber fractions, outperforming SBM. TP showed intermediate results, with protein degradability surpassing SBM at specific incubation times. Both WT and TP also exhibited higher small-intestinal protein digestibility than SBM. These results support the potential of tomato by-products as alternative protein sources, although larger *in vivo* trials are needed to validate their applicability.

Cavallini et al. evaluated the combination of reduced crude protein diets supplemented with rumen-protected amino acids and the substitution of cereals with fibrous by-products in eight multiparous Holstein dairy cows. Using four isoenergetic diets differing in protein level and starch source, the authors observed that lower-protein diets with amino acid supplementation reduced ruminal ammonia, improved nitrogen efficiency, and decreased water intake, without impairing dry-matter intake or rumination. However, the highest milk yield and fiber digestibility were associated with high-protein, high-cereal diets. These findings show that integrating rumen-protected amino acids and fibrous by-products can improve environmental efficiency, although productivity trade-offs must be considered.

Another emerging strategy involves the use of lignocellulosic biomass, which has low cost and does not compete with human edible resources. Ito et al. analyzed *in vitro* rumen fermentation and microbial responses using steamed and untreated woods, xylo-oligosaccharides, spent mushroom substrates, and conventional feeds. Steamed woods and xylo-oligosaccharides formed distinct microbial clusters dominated by *Succinivibrio* and *Selenomonas*, suggesting that specific hemicellulose-derived fractions can modulate rumen microbial communities and fermentation patterns. The results demonstrate the potential of lignocellulosic materials as sustainable feed ingredients, warranting further *in vivo* validation.

Beyond livestock, pet food production also competes with human food resources. Yuan et al. evaluated Japanese eel as a major ingredient in cat diets. Inclusion of eel improved coat condition, antioxidant capacity, calcium digestibility, and reduced serum triglycerides. The authors recommend 14% inclusion as an optimal level that balances nutritional benefits and sustainability.

Finally, for a proper ecological transition of the livestock sector, it is essential to ensure high-quality training for all operators involved, with a focus on environmental sustainability and on the appropriate use of by-products, agri-food co-products, and FFs' in animal feed. Diaz Vicuna et al. investigated, through an online survey, the perceptions of 136 Italian farm animal veterinarians regarding the adoption of FFs' and the influence of age and gender. The findings show that about 50% of veterinarians are aware of the definition of FFs' but are not currently recommending them. Almost 90% of respondents expressed a willingness to adopt FFs' as feed. Gender differences emerged: women considered the vitamin content and antioxidant properties of FFs' more important than men, while product availability was identified as a key factor for younger veterinarians. Participants willing to adopt FFs' as feed associated their positive attitudes with attributes such as digestibility, energy intake, and positive social implications.

In conclusion, the studies included in this Research Topic collectively demonstrate the potential of agro-industrial by-products, nutritional technologies, and innovative feed sources to reduce feed-food competition and enhance the sustainability of livestock systems.
